# Arginine vasopressin injection rescues delayed oviposition in *cyp19a1b* ^-/-^ mutant female zebrafish

**DOI:** 10.3389/fendo.2023.1308675

**Published:** 2023-12-08

**Authors:** Katherine Shaw, Chunyu Lu, Xiaochun Liu, Vance L. Trudeau

**Affiliations:** ^1^ Department of Biology, University of Ottawa, Ottawa, ON, Canada; ^2^ State Key Laboratory of Biocontrol, Institute of Aquatic Economic Animals and Guangdong Province Key Laboratory for Aquatic Economic Animals, School of Life Sciences, Sun Yat-Sen University, Guangzhou, China

**Keywords:** aromatase, brain, estrogen, neuropeptide, hormone, arginine vasopressin, sexual behavior, zebrafish

## Abstract

In zebrafish, estrogens produced in the ovaries via Cyp19a1a activity are required for both sexual differentiation of the ovary during early development as well as maintenance of the ovarian state during adulthood. The importance of Cyp19a1b that is highly expressed in the brain for female reproduction is still under study. We previously reported that female *cyp19a1b* ^-/-^ mutant zebrafish have significantly lower brain estradiol levels and impaired spawning behavior characterized by an increased latency to oviposition during dyadic sexual behavior encounters. In the current study, we provide evidence that the delayed oviposition in female *cyp19a1b* ^-/-^ mutants is linked to impaired arginine vasopressin (Avp) signaling. Droplet digital PCR experiments revealed that levels of the estrogen receptors, *avp*, and *oxytocin* (*oxt*) are lower in the hypothalamus of mutant females compared to wildtype fish. We then used acute intraperitoneal injections of Avp and Oxt, along with mixtures of their respective receptor antagonists, to determine that Avp can uniquely rescue the delayed oviposition in female *cyp19a1b* ^-/-^ mutants. Using immunohistochemistry, we demonstrated that Cyp19a1b-expressing radial glial cell (RGC) fibers surround and are in contact with Avp-immunopositive neurons in the preoptic areas of the brain. This could provide the neuroanatomical proximity for RGC-derived estrogens to diffuse to and activate estrogen receptors and regulate *avp* expression levels. Together these findings identify a positive link between Cyp19a1b and Avp for female zebrafish sexual behavior. They also suggest that the female *cyp19a1b* ^-/-^ mutant behavioral phenotype is likely a consequence of impaired processing of Avp-dependent social cues important for mate identification and assessment.

## 1 Introduction

Estrogens are critical for female reproduction (1). The final and rate limiting step for bioactive estrogen production involves the aromatase (Cyp19a1) enzyme that converts the aromatizable androgens, testosterone and androstenedione, into the bioactive estrogens, estradiol (E2) and estrone, respectively (2). Teleosts have two paralogs encoding aromatase, *cyp19a1a* and *cyp19a1b*, that are highly expressed in the ovaries and brain, respectively, due to distinct regulatory elements in their promoter regions (3, 4). Estrogens can exert diverse effects in the body by binding to different estrogen receptors (Esrs) that initiate various signalling pathways with tissue- and cell-specific effects (5). Gene expression can be regulated directly by nuclear Esrs (nEsrs) binding to estrogen response elements (EREs) in gene promoter regions or through indirect pathways involving protein-protein interactions with transcription factors such as the Activator protein 1 (Ap1) and the Specificity protein 1 (Sp1), when bound to their respective promoter elements (6). Though comparatively less studied, membrane-bound Esrs (mEsrs) have also been shown to affect the expression levels of estrogen-regulated genes through second messenger signalling pathways (7). Teleosts have three nEsrs named *estrogen receptor 1* (*esr1*), *estrogen receptor 2a* (*esr2a*), and *estrogen receptor 2b* (*esr2b;* 8), and the mEsr, *g-protein coupled estrogen receptor* (*gper*; 9).

A recent report indicated that *cyp19a1b* ^-/-^ mutant female zebrafish that have significantly lower brain E2 levels compared to wildtype (WT) females have altered female sexual behavior (10). Mutant *cyp19a1b* ^-/-^ females had delayed spawning behavior with WT males in sexual behavior assays compared to WT females. This delayed initiation of sexual behavior is reminiscent of the phenotype observed in aromatase knockout (Aro KO) mice that have reduced sexual behavior (11–15). The behavioral impairments in Aro KO mice are hypothesized to be linked to disrupted olfactory discrimination that reduces social recognition through effects on nEsrs and the nonapeptides, *oxytocin* (*oxt*) and *arginine vasopressin* (*avp*; 16–18). The overlapping behavioral impairments of the Aro KO mice and *cyp19a1b* ^-/-^ mutant zebrafish suggest potential similarities in the disruption of the signalling pathways underlying the behavioral phenotypes despite differences in cellular localization of brain aromatase. In mice, under normal conditions, aromatase is constitutively expressed in neurons (19). However, in teleosts, due to the presence of a cis-acting regulatory glial x responsive element in the *cyp19a1b* promoter region, aromatase is exclusively expressed in radial glial cells (RGCs; 3). Thus, the effects of RGC-derived estrogens on female sexual behavior are most likely to occur through E2 diffusing to bind Esrs in nearby neurons to affect signalling pathways involving estrogen-regulated genes important for behavior. There are several candidate genes known to affect zebrafish sexual behavior that could be linked to the female *cyp19a1b* ^-/-^ mutant behavioral phenotype.

An involvement of the nonapeptides in female teleost sexual behavior is strongly suggested by the findings of changes in brain nonapeptide levels across female reproductive states (20–27) and observations that exogenous E2 administration upregulates brain nonapeptide levels (28–31). The nonapeptides exert effects on reproduction through two main signalling pathways in the brain. Firstly, through a hypophysiotropic pathway involving magnocellular neurons in the preoptic area (POA) that project towards the pituitary gland for nonapeptide release into the circulatory system to regulate processes including osmoregulation, gonadal steroidogenesis, gametogenesis and parturition, amongst others (32–34). Secondly, through an encephalotropic pathway involving parvocellular and gigantocellular neurons that project to other brain regions including the telencephalon, prethalamus, hypothalamus, optic tectum, and the hindbrain (32, 33). The encephalotropic pathway is hypothesized to underly the neuromodulatory roles of the nonapeptides in affecting the salience of social information processing in the brain that can affect social recognition and behaviors (35). Indeed, recent findings from teleost studies have identified impairments in social recognition and behavior in *avp* ^-/-^ and *oxt* ^-/-^ mutant females that indicate important roles of these nonapeptides in female sexual behavior. For example, female *avp* ^-/-^ mutant zebrafish displayed reduced sexual behavior characterized by fewer quiver events produced, a measure of female sexual receptivity, when paired with WT males in sexual interactions (36). In another study, the mate preference of female Japanese medaka (*Oryzias latipes*) for familiar males was absent in *oxt* ^-/-^ and *oxytocin receptor 1* ^-/-^ (*oxtr1* ^-/-^) mutants suggesting reduced social recognition of mates compared to WT females during pairwise mating trials (37). Given the well conserved expression pattern of *cyp19a1b* in RGCs lining the teleost diencephalic ventricle (38), which is in close neuroanatomical proximity to the POA where high nonapeptide expression is observed (39), further study to characterize the interactions of *cyp19a1b* and nonapeptides in the female teleost brain in association with sexual behavior are well warranted.

Other important neuropeptides for female zebrafish sexual behavior are secretoneurin A (SNa) and secretoneurin B (SNb) that are derived from proteolytic processing of the precursor secretogranin 2a (Scg2a) and secretogranin 2b (Scg2b) proteins, respectively. Analysis of *scg2a* ^-/-^ and *scg2b* ^-/-^ frameshift mutant zebrafish lines demonstrated their critical importance for female sexual behavior, ovulation, oviposition, and fertility (40). Interactions between the secretogranergic system and Cyp19a1b have been previously identified in the teleost brain in regions important for sexual behavior. For example, *scg2a* and *scg2b* transcripts are expressed in numerous brain regions important for reproduction that lie close to the diencephalic ventricle (41) where *cyp19a1b* is highly expressed (38). A close neuroanatomical proximity between the soma of Scg2a-immunoreactive neurons and RGC fibres where Cyp19a1b is expressed was also identified in the POA of female goldfish (42). Intracerebroventricular injection of SNa downregulated *cyp19a1b* levels in the female goldfish telencephalon and hypothalamus, suggesting a regulatory pathway between SNa and neuroestrogen production (42). Together, these findings suggest potential interactions between the SNs and *cyp19a1b* that might be important for the regulation of female sexual behavior.

The purpose of this study was to investigate the mechanistic pathways through which *cyp19a1b* mutation in female zebrafish disrupts sexual behavior. Based on the findings from the various studies of Aro KO mice and the recent discoveries of the importance of the nonapeptides and secretoneurins for female zebrafish sexual behavior, we focused the study on identifying the potential interactions between *cyp19a1b*, the nEsrs and these neuropeptides. We first used droplet digital polymerase chain reaction (ddPCR) experiments to identify significantly lower levels of *avp* and *oxt* in the hypothalamus of female *cyp19a1b* ^-/-^ mutants. We then conducted acute injections of the nonapeptides and observed that Avp uniquely rescued the delayed oviposition in female *cyp19a1b* ^-/-^ mutants and this effect was blocked with administration of an arginine vasopressin receptor 1a (Avpr1a) antagonist. Using immunohistochemistry, the preoptic areas were identified as regions with close neuroanatomical proximity between Cyp19a1b-expressing RGCs and Avp-immunopositive neurons in the female zebrafish brain. Together, our findings demonstrate that the impaired sexual behavior in female *cyp19a1b* ^-/-^ mutant zebrafish is likely a consequence of disrupted estrogenic regulation of *avp* in preoptic areas and that these behavioral effects can be rescued by acute Avp administration in adult fish.

We have adopted the guidelines of the Zebrafish Information Network (ZFIN, www.zfin.org) for nomenclature to designate teleost genes and their protein counterparts. Of note, the recently determined zebrafish nomenclature for *avp*/Avp and *oxt*/Oxt contrasts the historically used nomenclature of *avt*/Avt and *ist*/Ist. This change highlights the homologous nature of the nonapeptide genes and their protein counterparts rather than the differences in the teleost and mammalian amino acid compositions.

## 2 Materials and methods

### 2.1 Experimental animals

Wildtype, *cyp19a1b* ^-/-^ mutant female (43), and *cyp19a1b*-GFP transgenic (Tg(*cyp19a1b*-GFP); 44) zebrafish were raised in the University of Ottawa Aquatics Facility. Fish were housed in 10-L tanks of dechloraminated recirculating water maintained at 28°C on a 14:10 light:dark cycle, and fed twice daily at 11h00 and 15h00. All fish tested in this experiment were between the ages of 5-11 months post-fertilization and had no previous experience in the sexual behavior assay; with males and females separated for at least two weeks prior to testing.

### 2.2 Total RNA extraction and cDNA synthesis

For gene expression measurements, the telencephalon and hypothalamus were selected for study because they contain many key brain areas for regulating sexual behavior and reproduction (45). Female WT and *cyp19a1b* ^-/-^ mutant fish (n=6-9 per group) were euthanized in an ice water bath at two time points corresponding to during (10h00) and outside (14h00) the timing of zebrafish reproductive behavior. The brains were immediately removed, and the telencephalon was dissected at its connections to the olfactory bulb and hypothalamus and placed into a 1.5 mL RNase-free Eppendorf tubes on dry ice. The brains were then inverted to dissect the hypothalamus region including the ventrolateral extending hypothalamic lobes and diencephalon tissue ventral to the optic tectum and placed into separate 1.5 mL RNase-free Eppendorf tubes on dry ice. This dissection method produces telencephalon samples containing the dorsal and ventral telencephalon regions as well as the anterior preoptic area encompassing the parvocellular preoptic neurons, whilst the hypothalamus samples contain the posterior preoptic area including the magnocellular and gigantocellular neurons and surrounding hypothalamic regions. Total ribonucleic acid (RNA) was isolated from samples using Trizol (Thermo Fisher Scientific, Waltham, MA, USA, cat# 15596018) according to the manufacturer’s instructions with a few modifications. Briefly, 50 µL Trizol was added to each tube and tissues were homogenized using a pestle with intermittent vortexing. Samples were incubated for 5 mins at room temperature and then 10 µL chloroform was added to each tube, vortexed, and allowed to incubate for 3 mins. Following incubation, samples were centrifuged for 5 mins at 12,600 rpm at 4°C, the supernatant was carefully transferred to a new labelled RNase-free 1.5 mL tube and 25 µL isopropanol was added to each tube, and samples were vortexed. The tubes were incubated for 10 mins at room temperature and then centrifuged for 30 mins at 12,600 rpm at 4°C. The isopropanol was removed, and pellets were rinsed with 50 µL of 75% ethanol (EtOH) followed by centrifugation for 5 mins at 12,600 rpm at 4°C. The EtOH was then removed from tubes and the rinse step was repeated. The tubes were then left open for 2 mins in a fumehood to dry the samples. Samples were resuspended in 20 µL RNase-free ddH_2_O and incubated for 10 mins at 60°C on a heat block. The samples were then vortexed and placed on ice before quality assessment. Total RNA concentration as well as 260/280 and 260/230 absorbance measurements were assessed using a spectrophotometer (Thermo Fisher Scientific, NanoDrop 2000). RNA integrity was verified using gel electrophoresis (1% weight/volume agarose). RNA was stored at -80°C until further usage. Total complementary DNA (cDNA) was synthesized using the iScript Reverse Transcription Supermix (Bio-Rad, Hercules, CA, USA, cat# 1708841), according to the manufacturer’s instructions. Synthesis of cDNA used 600 ng of total RNA from telencephalon and hypothalamus samples and cDNA was stored at -20°C.

### 2.3 Primer design

Primers for gene expression analysis were designed using either NCBI Primer Blast Software (https://www.ncbi.nlm.nih.gov/tools/primer-blast/) or obtained from previous gene expression analysis studies (40, 41, 46). All primers were supplied by Integrated DNA Technologies (Coralville, IA, USA) and the specificity of primers for the genes of interest were confirmed by sanger sequencing of PCR products (Génome Québec, Montréal, QC, Canada) and sequences were matched to the zebrafish genome using the NIH Basic Local Alignment Search Tool (https://blast.ncbi.nlm.nih.gov/Blast.cgi). Appropriate annealing temperatures and cDNA dilutions for ddPCR analysis were identified by performing thermal gradients (51-63°C) with cDNA collected from telencephalon and hypothalamus (1:2 – 1:320 dilution) samples. Primer sequences, annealing temperatures, and amplicon sizes are presented in **Table 1**.

**Table 1:** Primer pairs and experimental conditions for gene expression analyses of telencephalon and hypothalamus samples.

Gene Name	Gene Abbreviation	Primer	Annealing Temperature (°C)	Amplicon Size (bp)
*estrogen receptor 1*	*esr1*	F:CACATCAGACACATGAGCAACAA R:CTGAAGACTGGAACCGCTGA	55.7	121
*estrogen receptor 2a*	*esr2a*	F:GCCTGCCGACTCCGAAA R:TTGTTGGTAGCTGCTACGATCTCT	55.7	77
*estrogen receptor 2b*	*esr2b*	F: CGACTCCGCAAGTGCTATGAA R: ACGATGTCGAGCACCTCGAT	55.7	83
*arginine vasopressin*	*avp*	F:AGGTCTGCATGGAAGAGGAG R:CTGCCTTCAGGACAGTCTGG	60.7	146
*oxytocin*	*oxt*	F:GATCTGCTGCTGAAGCTCCT R:TACAAAAGTGGGTGGCGAGT	60.7	134
*secretogranin 2a*	*scg2a*	F:CAGGACGTACGGGTTATGCT R:GCGTTGGTCTTTGGTTTTGT	60.7	138
*secretrogranin 2b*	*scg2b*	F:AAACAAAGCTCCGAGCAAAA R:AACTGGTGTCGGGATACTCG	55.7	116
*tata-binding protein*	*tbp*	F:TACCCACCAGCAGTTTAGCA R:TCTAACCTTGGCACCTGTGA	60.7	130

### 2.4 Droplet digital PCR protocol

The ddPCR procedures including experiment set-up, droplet generation, and transfer of emulsified samples to PCR plates, were performed according to the manufacturer’s protocols (Bio-Rad, QX200 Droplet Digital PCR system). In brief, at the start of the experiment, telencephalon or hypothalamus cDNA samples were diluted to the appropriate concentration determined for each primer pair. The reaction mixtures were prepared, each containing 11.5 µL EvaGreen ddPCR Supermix (Bio-Rad, cat# 1864034), 0.23 µL of 10 µM gene-specific forward and revere primers, 6.04 µL nuclease-free water, and 5 µL of template cDNA at primer-specific dilution. 20 µL of the sample reactions were then carefully transferred into the wells of a droplet generation cartridge (Bio-Rad, cat# 1864008) along with droplet generation oil (Bio-Rad, cat# 1864006) to the corresponding oil wells. The cartridge was sealed with a gasket (Bio-Rad, cat# 1863009) and then inserted into a droplet generator (Bio-Rad, model# QX200 Droplet Generator) for droplet generation. The emulsified samples were then carefully transferred to the wells of a 96-well ddPCR plate (Bio-Rad, cat# 12001925) and sealed with a heat foil (Bio-Rad, cat# 1814040). The PCR reactions were performed using a thermal cycler (Bio-Rad, model# C1000 Touch Thermal Cycler) with the cycle conditions: (95°C for 5 mins), 49 cycles of ((95°C for 30 secs), (annealing temperature for 1 min), (72°C for 30 secs), with a ramp rate of -2°C/sec), followed by (4°C for 5 mins), (90°C for 5 mins), and finally (12°C for 5 mins). The fluorescence of droplets was measured using a droplet reader (Bio-Rad, model# QX200 Droplet Reader) and data were analyzed using QuantaSoft Analysis Pro software (Bio-Rad, v1.0.596).

### 2.5 Brain estradiol enzyme-linked immunosorbent assays

Validated brain steroid extraction (47) and E2 quantification (10) using ELISA test kits (Cayman Chemical, cat# 501890) have been described previously. In brief, brains were collected at two time points corresponding to the morning at the time of lights on when zebrafish are sexually active (10h00) and at a later midpoint time in the light cycle when fish are not sexually active (14h00; n=4-5 per group). At each time point, both WT and *cyp19a1b* ^-/-^ mutant female fish were euthanized in an ice water bath, and brains were immediately dissected and placed into individual labelled 1.5 mL Eppendorf tubes. Homogenization buffer (150 µL per brain) was added to each tube and tissues were then individually sonicated for steroid release into solution. Tubes were centrifuged (4°C, 13,200 rpm, 10 mins) and the supernatant was then carefully removed and transferred to a new labelled tube and evaporated to dryness (Labconco Centrivap Centrifugal Vacuum Concentrator, model# 7810014, 45°C, 1 hr), then stored at 4°C overnight. The next day, samples were resuspended with 100 µL resuspension buffer added to each tube, vortexed, and tubes were sonicated in a water bath for 15 mins. Following resuspension, samples were run on C-18 solid-phase extraction columns (47) with a final elution volume of 120 µL. The eluted samples were then evaporated to dryness (45°C, 3 hrs). The evaporated residue was then resuspended in 200 µL ELISA buffer for 24 hrs at 4°C with intermittent vortexing prior to testing. Samples were run in duplicate and only samples with intra-assay coefficient of variations < 10% were used in analyses.

### 2.6 Characterizing female *cyp19a1b* ^-/-^ mutant sexual behavior following peptide injection

The methods used in this experiment are similar to those previously described (10, 47). For all trials, a *cyp19a1b* ^-/-^ mutant female was size-matched with a male WT fish (within 2 mm BL, < 10% BL difference). All *cyp19a1b* ^-/-^ mutant females tested were derived from a parental strain to ensure identical genetic backgrounds and raised in the same environmental conditions for assessing the effects of treatment on spawning behavior. The WT males tested were similarly derived from a parental strain and raised in the same environmental conditions. The evening before each experiment, paired fish were transferred to a 1-L testing tank containing an insert at the bottom for egg collection and a divider in the middle of the tank to keep the male and female separate before testing. The testing pair was allowed to acclimate overnight in the Zebracube (Viewpoint Behavior Technology, Inc.) with the camera present (either a Panasonic 16GB HC-V700M Full HD camcorder or a Canon VIXIA HF R800 camcorder). The following morning at 9h00, the pair was transferred to a new 1-L testing tank containing clean system water. The female was immediately removed and anesthetized with Tricaine (Syndel, cat# 02168510) according to University of Ottawa zebrafish husbandry procedures, then weighed on an analytical balance, and transferred to a moist sponge with an insert cut for positioning the female for injection. Females (n=15 per group) were intraperitoneally injected with dosages based on bodyweight (bw) using a 32-gauge syringe (Hamilton Company, Reno, NV, USA, cat# CAL80308) with either 1) Ringer’s solution (pH 7), 2) Oxt (0.5 µg/g bw), 3) Avp (0.5 µg/g bw), 4) mix of 0.5 µg/g bw Avp + 5 µg/g bw Manning Compound (an Avpr1a antagonist), or 5) mix of 0.5 µg/g bw Avp + 5 µg/g bw L-368,899 (an Oxtr antagonist). The zebrafish Oxt and Avp peptides were prepared *in house* by Fmoc/tBu solid-phase peptide synthesis, and sequences were confirmed by mass spectrometry. The peptide receptor antagonists, Manning Compound (Bio-Techne Canada, Toronto, ON, Canada, cat# 3377) and L-368,899 (Bio-Techne Canada, cat# 2641) were dissolved in water according to manufacturer’s instructions. Following injection, the female was immediately transferred to a new housing tank containing clean system water to recover for 5 mins before being transferred back to the testing tank. The divider was then removed, and video recording started. All filming began within 15 mins of lights on (9h00). The pairs were allowed to interact for 60 mins to determine whether the peptide injections could rescue the delayed time to the first oviposition event observed in the female *cyp19a1b* ^-/-^ mutants (10). Videos were coded by date and viewed by an observer blind to the treatment groups using VLC media player (https://www.videolan.org/) to record the time to the first oviposition event.

### 2.7 Immunohistochemistry

Fish (n=20) from the Tg(*cyp19a1b*-GFP) line were euthanized using an ice water bath and carefully positioned under a dissection microscope for the careful removal of scales on the skull cap using dissecting tweezers to expose the brain. Fish heads were then dissected away from the body and placed immediately in a falcon tube containing 4% paraformaldehyde in phosphate-buffered saline (PBS; pH 7.4) for 24 hrs fixation on an orbital shaker at room temperature. Fixed samples were then washed with PBS (3 x 20 mins), following which the heads were placed in 0.5 M ethylenediaminetetraacetic acid for 7 days for decalcification. Following decalcification, the samples were then successively dehydrated for 30 mins each in 30%, 50%, 70%, 80% and finally 99% EtOH. Samples were cleared by 2 x 1 hr incubations in 100% xylene, incubated for 1 hr in melted paraffin at 60°C, and then transferred to new melted paraffin and left overnight at 60°C. The next day, the samples were embedded in a cassette containing newly melted paraffin with heads oriented for transverse sectioning and allowed to cool. The embedded heads were sectioned at 15 µm thickness using a motorized microtome (Leica Biosystems, model# RM2255). Sample sections were spread on a 40°C water bath to remove wrinkles and then mounted on Superfrost Plus slides (Thermo Fisher Scientific, cat# 22-037-246). Slides were allowed to air dry and then stored in a slide box at 4°C prior to use.

All wash steps were conducted using glass Coplin jars (Ted Pella, Inc., Redding, CA, USA, cat# 432-1). Slides were first heated for 1 hr at 60°C on a slide warmer to improve tissue adherence to the slides during wash steps. The slides were then deparaffinized by xylene washes (2 x 20 mins) and rehydrated using a series of washes including 2 x 100% EtOH, 2 x 95% EtOH, 1 x 70% EtOH, 1 x PBS, each for 10 mins. The slides were then transferred to Coplin jars containing 0.01 M sodium citrate (pH 6.0) and placed in a 90°C water bath for 30 mins for antigen retrieval. Following this, slides were allowed to cool and washed in PBS (2 x 5 mins), then incubated in blocking buffer (1% bovine serum albumin in 0.3% Triton PBS) for 30 mins. Slides were then placed in a humid chamber at 4°C to incubate overnight with the primary antibodies: anti-Avp (rabbit, Immunostar, Hudson, WI, USA, cat# 20069) and anti-GFP (chicken, Abcam, Cambridge, UK, cat# ab13970), diluted 1:500 in blocking buffer. The anti-Avp antibody was validated and found to be specific for teleost Avp previously in an electric fish (48) and confirmed for zebrafish in this study (see below). The following day, the slides were washed in PBS (2 x 10 mins), and subsequently incubated for 2 hrs in a dark humid chamber at room temperature with the secondary antibodies: donkey anti-rabbit IgG (H+L) Alexa Fluor 680 (Invitrogen, Waltham, MA, USA, cat# A10043) and goat anti-chicken IgY (H+L) Alexa Fluor 594 (Invitrogen, cat# A11042), diluted 1:500 in PBS. The next day, slides were washed in the dark in PBS (2 x 10 mins), then incubated in 200 µL Hoechst solution (1:10,000, Invitrogen, cat# H3570) in a dark chamber for 8 mins, followed by PBS washes (3 x 5 mins) in the dark, and mounted with SlowFade Diamond Antifade Mountant (Invitrogen, cat# S36963) and a cover slip (VWR, Radnor, PA, USA, cat# 48393-221). Slides were stored in the dark until visualization. Slides were imaged using an Olympus confocal microscope (Olympus, Richmond Hill, ON, Canada, model #FV1000) and FV10-ASW-4.2 Viewer software.

### 2.8 Antibody characterization

The Avp antibody has been previously validated for use in the teleost *Brachyhypopomus gauderio* (48). To validate the antibody for use in zebrafish, sections were incubated with anti-Avp (1:500) pre-adsorbed for 24 hrs with an excess of zebrafish Avp (10 µM). No Avp labelling was observed in the sections demonstrating primary antiserum specificity. Separate sections were incubated with anti-Avp (1:500) pre-adsorbed for 24 hrs with an excess of zebrafish Oxt (10 µM). Avp labelling was unaffected by pre-adsorption with Oxt, demonstrating antibody specificity for the Avp antigen. Control sections where the primary or secondary antibodies were omitted did not display any labelling.

### 2.9 Statistical analyses

Statistical analyses were conducted using Graphpad Prism v9 (GraphPad Software, Inc.). For both gene expression data and E2 measurements, normality of residuals and homoscedasticity were assessed using Shapiro-Wilk tests and Spearman’s tests for heteroscedasticity, respectively. Data were then analyzed in Two-Way ANOVA tests followed by Tukey’s multiple comparisons tests with the Holm-Šídák method used to correct for multiple comparisons. Gene expression data that were non-normally distributed were either log- or square root-transformed, and data that could not be normalized through transformation were grouped by genotype and assessed using Mann Whitney tests with the Holm-Šídák method to correct for multiple comparisons. The absolute transcript levels of target genes in the telencephalon and hypothalamus were normalized to *tata-binding protein* (*tbp*), an internal housekeeping gene that is not estrogen-regulated (49). There were no significant differences observed in *tbp* levels across genotype groups or time points in the study confirming the appropriate usage of *tbp* as a housekeeping gene. Gene expression levels are presented as fold change relative to WT female levels at 10h00 with individual points and means or median values displayed. For the behavioral rescue experiments, normality and homoscedasticity were assessed using Shapiro-Wilk and Levene’s tests, respectively. If a pair did not spawn eggs in the 60-min test period, a maximum latency of 60 mins was assigned. Data were analyzed using a Kruskal-Wallis test followed by Dunn’s multiple comparisons tests for pairwise comparisons. Data are presented as medians, with values for individual pairs displayed. For all data, significance was defined at p < 0.05 and all significance tests were assessed as two-tailed.

## 3 Results

### 3.1 Decreased expression levels of reproductive neuroendocrine genes in the telencephalon and hypothalamus of *cyp19a1b* ^-/-^ mutant females

#### 3.1.1 Telencephalon

Two-Way ANOVA was used to assess the effects of time (10h00 and 14h00) and genotype (*cyp19a1b* ^-/-^ mutant and WT) on gene expression levels in the telencephalon.

**Figure 1:**
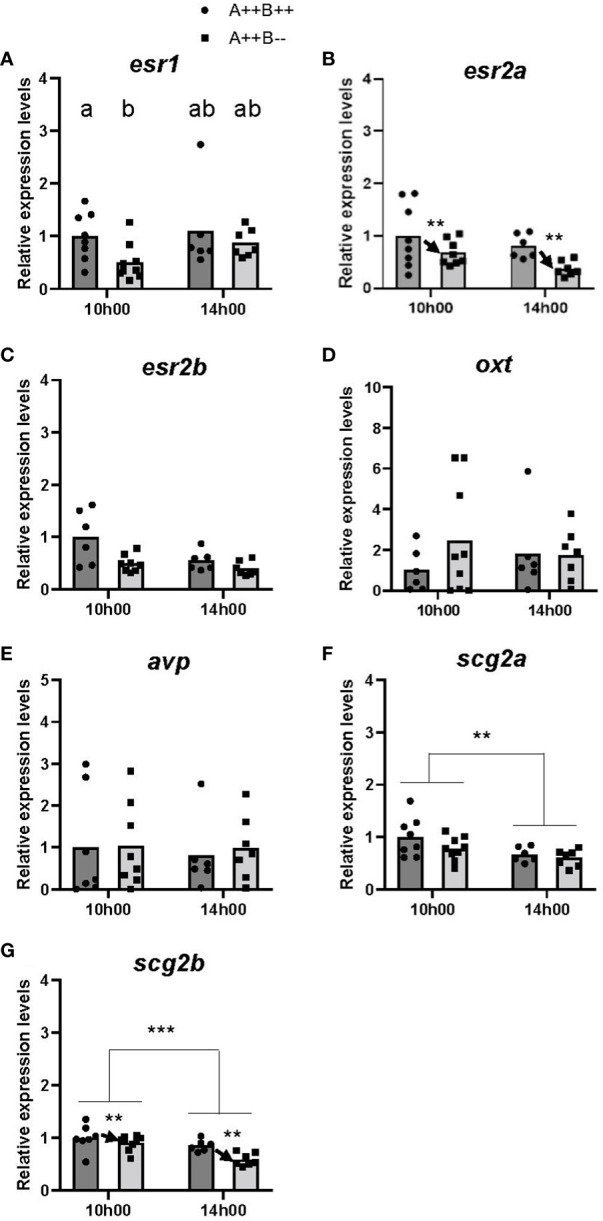
Gene expression in the telencephalon of adult wildtype (A++B++) and cyp19a1b^-/-^ mutant (A++B–) female zebrafish in the morning (10h00) and afternoon (14h00). The abundance of mRNAs (copies/µL) for all genes measured was normalized by tata-binding protein levels within the sample and data are plotted as fold change relative to the wildtype female levels at 10h00. Parametric data were analyzed by Two-Way ANOVA tests followed by Tukey’s multiple comparisons tests and individual values are plotted with bars representing mean values **(A, B, D, F, G)**, whilst non-parametric data were analyzed using multiple Mann-Whitney U tests with a Holm-Šídák correction for multiple comparisons and plotted as individual points with bars representing median values **(C, E)**. Error bars are not displayed for clarity. Significant main effects of time (horizontal bars) and genotype (arrows) are indicated by asterisks (** p<0.01; *** p<0.001). When there is a statistically significant interaction effect, means with different letters a-b represent statistically significant differences (p<0.05).

There were small differences in the expression levels of the Esrs between the telencephalon of female WT and *cyp19a1b* ^-/-^ mutants. While there were no significant main effects of time (F(1,25)=0.9293, p=0.3443) or genotype (F(1,25)=2.668, p=0.1149) on *esr1* levels, there was a statistically significant time X genotype interaction (F(1,25)=5.554, p=0.0266). *Post-hoc* tests revealed that the telencephalon of WT females had 1.9 times higher *esr1* levels compared to *cyp19a1b* ^-/-^ mutant females at 10h00 (p=0.0217; **Figure 1A**). There were no other significant interactions. A significant main effect of genotype (F(1,25)=8.662, p=0.0069), but not of time (F(1,25)=3.400, p=0.0771) or time X genotype interaction (F(1,25)=2.375, p=0.1358), on *esr2a* levels was observed. The telencephalon of WT females had 1.7 times higher *esr2a* levels compared to *cyp19a1b* ^-/-^ mutant females (**Figure 1B**). For *esr2b* levels, there were no significant differences in *esr2b* levels at either 10h00 (U(6,8)=21.00, p=0.2991) or 14h00 (U(5,6)=9.00, p=0.1925; **Figure 1C**).

No significant main effects were observed of time (F(1,24)=0.2294, p=0.6363), genotype (F(1,24)=0.5074, p=0.4831), or time X genotype interaction (F(1,24)=0.3781, p=0.5444) on *oxt* levels (**Figure 1D**). There were no significant differences in *avp* levels at either 10h00 (U(7,8)=34.00, p=0.8884) or 14h00 (U(5,6)=17.00, p=0.8618; **Figure 1E**).

There were statistically significant main effects of time for both *scg2a* (F(1,26)=7.717, p=0.0100) and *scg2b* (F(1,25)=14.21, p=0.0009) levels in the telencephalon. Levels of *scg2a* and *scg2b* were 1.4 and 1.3 times higher, respectively, at 10h00 compared to 14h00 (**Figures 1F, G**). There was also a significant main effect of genotype (F(1,25)=8.833, p=0.0065) on *scg2b* levels. The telencephalon of WT females had 1.2 times higher *scg2b* levels compared to *cyp19a1b* ^-/-^ mutant females (**Figure 1G**). There was no significant main effect of genotype on *scg2a* levels (F(1,26)=2.501, p=0.1258) or time X genotype interaction on *scg2a* levels (F(1,26)=0.4523, p=0.5072) or *scg2b* levels (F(1,25)=2.835, p=0.1047).

#### 3.1.2 Hypothalamus

Two-way ANOVA was performed to assess the effects of time (10h00 and 14h00) and genotype (*cyp19a1b* ^-/-^ mutant and WT) on gene expression levels in the hypothalamus.

**Figure 2:**
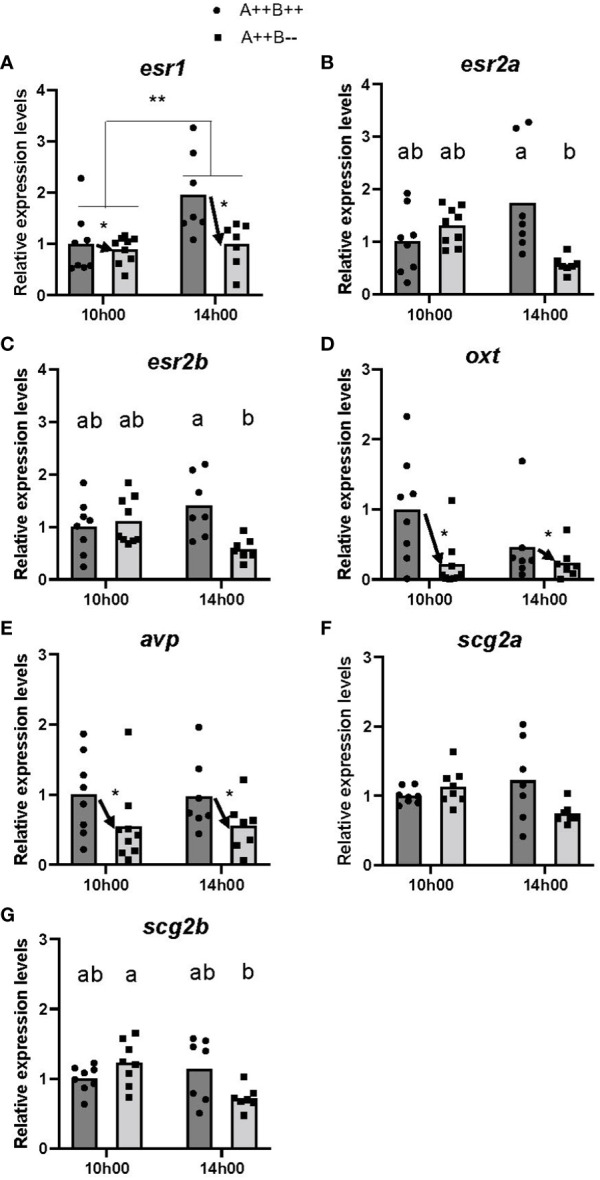
Gene expression in the hypothalamus of adult wildtype (A++B++) and cyp19a1b^-/-^ mutant (A++B–) female zebrafish in the morning (10h00) and afternoon (14h00). The abundance of mRNAs (copies/µL) for all genes measured were normalized by tata-binding protein levels within the sample and data are plotted as fold change relative to the wildtype female levels at 10h00. Parametric data were analyzed by Two-Way ANOVA tests followed by Tukey’s multiple comparisons tests and individual values are plotted with bars representing mean values **(A–E, G)**, whilst non-parametric data were analyzed using multiple Mann-Whitney U tests with a Holm-Šídák correction for multiple comparisons and plotted as individual points with bars representing median values **(F)**. Error bars are not displayed for clarity. Significant main effects of time (horizontal bars) and genotype (arrows) are indicated by asterisks (* p<0.05; ** p<0.01). When there is a statistically significant interaction effect, means with different letters a-b represent statistically significant differences (p<0.05).

There were significant differences in *esr* levels between the hypothalamus of WT and *cyp19a1b* ^-/-^ mutants. There were significant main effects of time (F(1,26)=8.967, p=0.0060) and genotype (F(1,26)=5.475, p=0.0272) on *esr1* levels. Levels of *esr1* were 1.6 times higher at 14h00 compared to 10h00 (**Figure 2A**), and WT females had 1.5 times higher *esr1* levels compared to *cyp19a1b* ^-/-^ mutant females (**Figure 2A**). There was no significant time X genotype interaction (F(1,26)=3.758, p=0.0635). No significant main effects of time on *esr2a* (F(1,25)=2.139, p=0.1560) or *esr2b* (F(1,27)=0.4068, p=0.5290) levels, or main effects of genotype on *esr2a* (F(1,25)=0.6108, p=0.4418) or *esr2b* (F(1,27)=3.782, p=0.0623) levels were observed. However, there were significant time X genotype interactions for *esr2a* (F(1,25)=10.65, p=0.0032) and *esr2b* (F(1,27)=9.103, p=0.0055) levels. *Post-hoc* tests revealed that the hypothalamus of WT females had 3.1 times higher *esr2a* levels compared to *cyp19a1b* ^-/-^ mutant females at 14h00 (p=0.0076; **Figure 2B**). Levels of *esr2b* were 2.4 times higher in the hypothalamus of WT females compared *cyp19a1b* ^-/-^ mutant females at 14h00 (p=0.0120; **Figure 2C**). No other significant interactions were evident.

There were large differences in *oxt* and *avp* levels in the hypothalamus of WT and *cyp19a1b* ^-/-^ mutant females. Significant main effects of genotype on both *oxt* (F(1,27)=7.300, p=0.0118) and *avp* (F(1,27)=6.848, p=0.0144) levels were observed. The hypothalamus of WT females had 3.2 and 1.8 times higher *oxt* and *avp* levels, respectively, compared to *cyp19a1b* ^-/-^ mutant females (**Figures 2D, E**). There were no significant main effects of time on *oxt* (F(1,27)=0.0019, p=0.9656) or *avp* (F(1,27)=0.0848, p=0.7731) levels, or time X genotype interactions on *oxt* (F(1,27)=1.373, p=0.2515) or *avp* (F(1,27)=0.0083, p=0.9281) levels.

No significant differences in *scg2a* levels at either 10h00 (U(6,7)=22.00, p=0.3282) or 14h00 (U(6,7)=11.00, p=0.1852; **Figure 2F**) were observed. There was a significant time X genotype interaction on *scg2b* levels (F(1,26)=6.704, p=0.0156), with *post-hoc* tests revealing that *cyp19a1b* ^-/-^ mutant females had 1.7 times higher *scg2b* levels at 10h00 compared to 14h00 (p=0.0153; **Figure 2G**). There were no other significant interactions and no main effects of time (F(1,26)=4.097, p=0.0537) or genotype (F(1,26)=0.8427, p=0.3671) on hypothalamic *scg2b* levels.

### 3.2 Brain estradiol content fluctuates between the morning and afternoon

**Figure 3:**
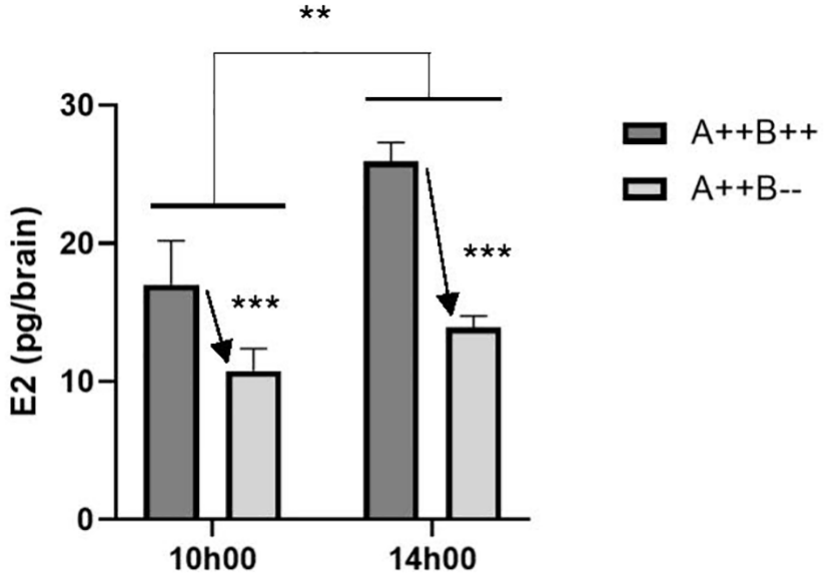
Estradiol (E2) levels in the brains of adult wildtype (A++B++) and cyp19a1b^-/-^ mutant (A++B–) female zebrafish in the morning (10h00) and afternoon (14h00). Data were analyzed using a Two-Way ANOVA followed by Tukey’s multiple comparisons tests. Significant main effects of time (**, p=0.0072) and genotype (*** with arrow; p=0.0003) are indicated. There was no significant interaction effect. Data are plotted as means + standard error of the mean (n=4-5 per group).

There was a significant main effect of time on brain E2 levels (F(1,13)=10.14, p=0.0072). Brain E2 levels were 1.4 times higher at 14h00 compared to 10h00 (**Figure 3**). There was also a significant main effect of genotype (F(1,13)=23.04, p=0.0003). WT brains had 1.7 times higher E2 levels compared to *cyp19a1b* ^-/-^ mutant brains (**Figure 3**). There was no significant time X genotype interaction (F(1,13)=2.291, p=0.1541).

### 3.3 Arginine vasopressin injection rescues the behavioral phenotype in *cyp19a1b* ^-/-^ mutant females

**Figure 4:**
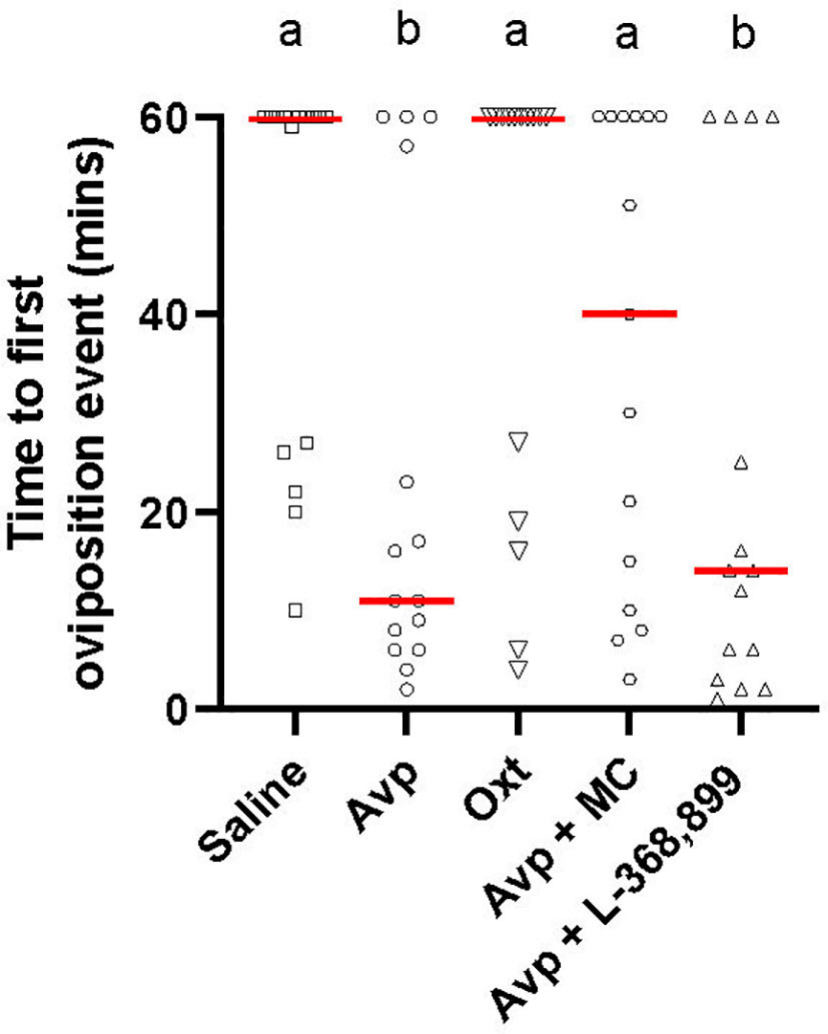
Time to first oviposition event during zebrafish pairwise mating trials with female cyp19a1b^-/-^ mutant fish intraperitoneally injected with nonapeptides and mixed nonapeptides with receptor antagonists (n=15 per group). Significant differences were assessed using a Kruskal-Wallis test followed by a Dunn’s multiple comparisons test. Individual data points are displayed with red bars representing median values. Different letters a-b represent statistically significant differences. Statistical significance is defined at p<0.05.

There was a significant difference in the time to the first oviposition event amongst the *cyp19a1b* ^-/-^ mutant female groups (H(4)=14.87, p=0.005; **Figure 4**). Pairwise comparisons revealed that both Avp (p=0.0221) and Avp + L-368,899 (p=0.0155) groups took significantly less time to the first oviposition event compared to the saline-injected group. Saline-injected *cyp19a1b* ^-/-^ mutant females took 5.5 and 4.3 times longer to the first oviposition event compared to Avp- and Avp + L-368,899-injected *cyp19a1b* ^-/-^ mutant females, respectively. There were no significant differences in the time to the first oviposition event in the Avp + Manning Compound (p=0.7986) or Oxt (p>0.9999) groups compared to saline-injected fish.

### 3.4 Cyp19a1b-immunopositive radial glial cell fibres contact arginine vasopressin cells in preoptic areas

**Figure 5:**
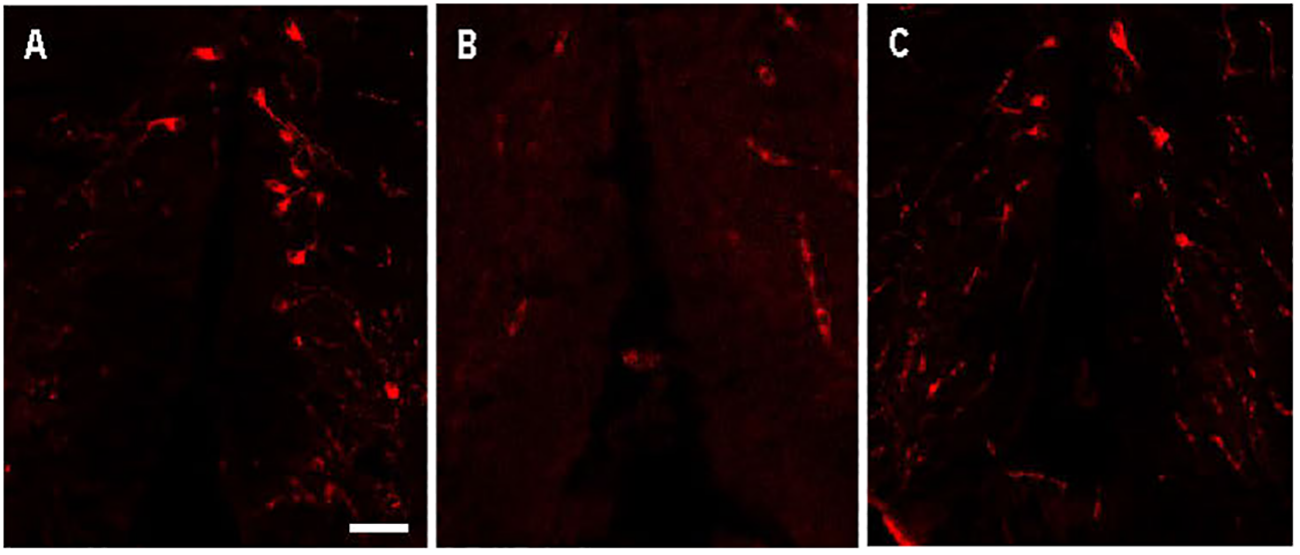
Arginine vasopressin (Avp) immunolabelling in pre-adsorption tests with Avp and Oxytocin (Oxt) in a transverse section of a female wildtype zebrafish preoptic area. The confocal images show Avp-immunopositive cell bodies surrounding the diencephalic ventricle **(A)**. No Avp-immunopositive neurons were present in sections pre-adsorbed with Avp **(B)**. Avp immunolabelling was unaffected in sections pre-adsorbed with Oxt **(C)**. Scale bar=20 µm.

**Figure 6:**
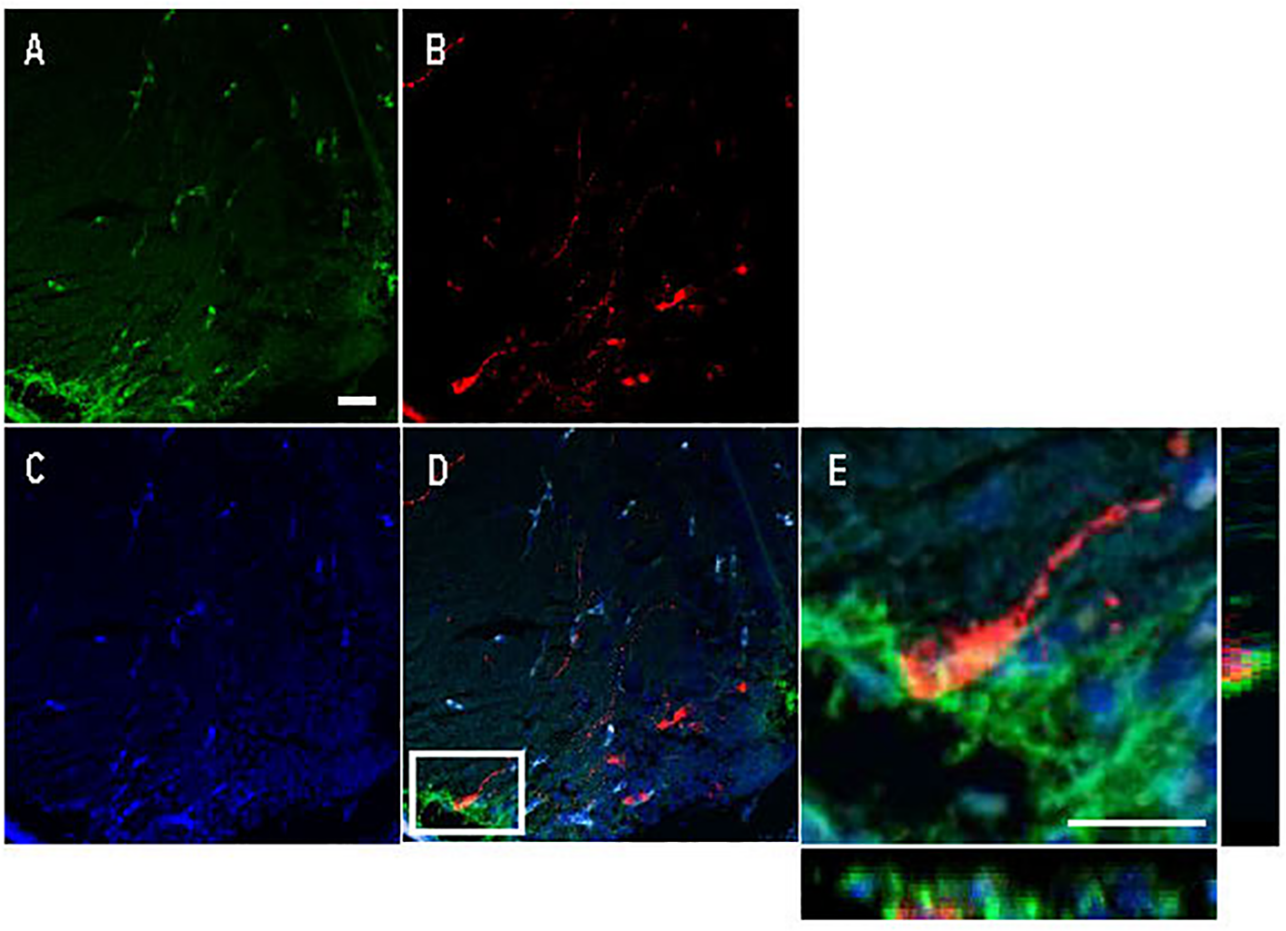
Double immunofluorescence against Cyp19a1b (green) and Arginine vasopressin (Avp; red) in a female Tg(cyp19a1b-GFP) zebrafish anterior preoptic area. In this transverse brain section, Cyp19a1b-positive radial glial cells fibres lining the peripheral layer of the ventral telencephalon **(A)** surround an Avp-immunopositive parvocellular cell body **(B)**. The nuclear stain Hoescht (blue) is also shown **(C)**. Single slice scanning view of the boxed area in panel D shows Cyp19a1b-positive fibres in contact with the Avp-immunopositive cell body **(E)**. Scale bar=20 µm.

**Figure 7:**
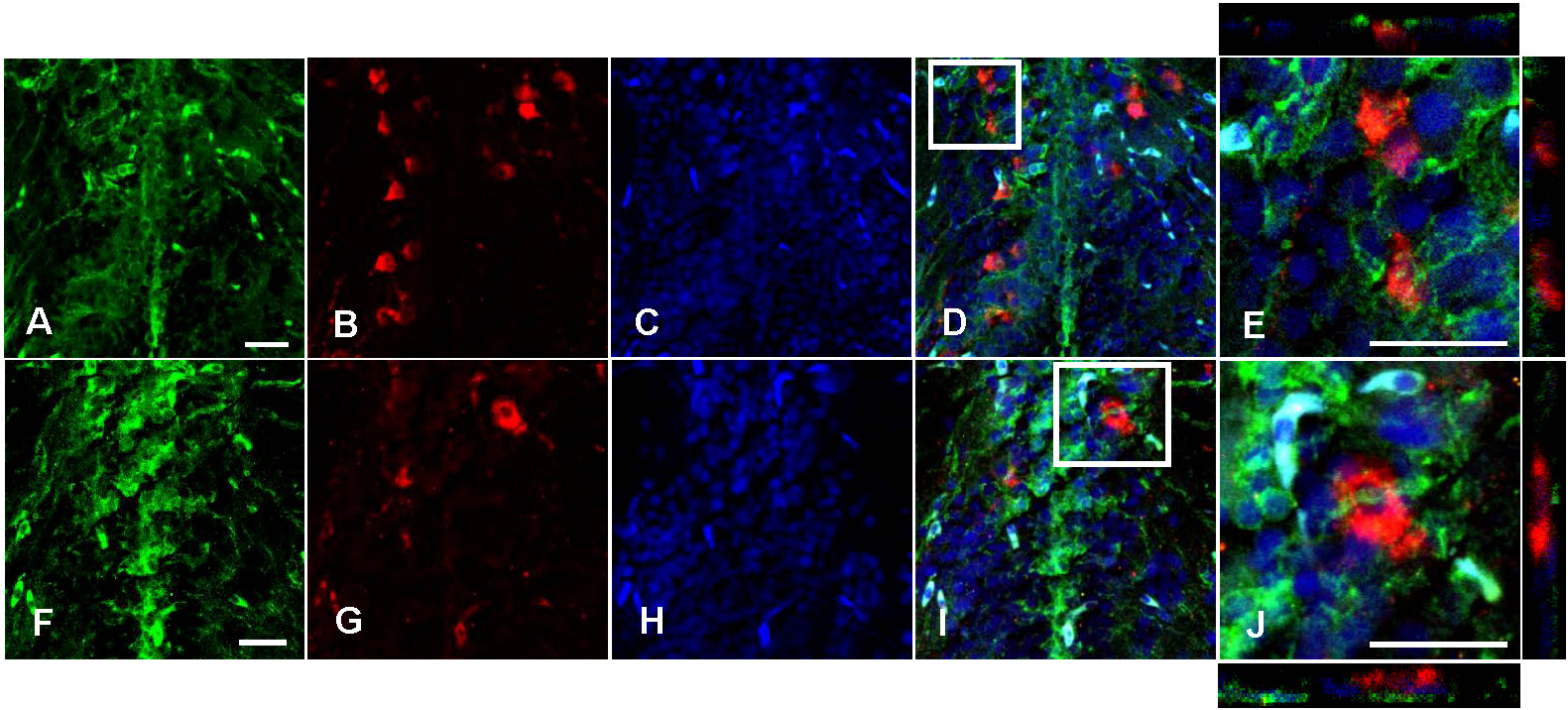
Double immunofluorescence against Cyp19a1b (green) and Arginine vasopressin (Avp; red) in a female Tg(cyp19a1b-GFP) zebrafish posterior preoptic area. In this transverse brain section, Cyp19a1b-positive radial glial cell fibres **(A, F)** surround Avp-immunoreactive magnocellular **(B)** and gigantocellular **(G)** cell bodies. The nuclear stain Hoescht (blue) is also shown **(C, H)**. Single slice scanning view of the boxed areas in **(D, I)** show Cyp19a1b-positive fibres in contact with the Avp-immunopositive cell bodies **(E, J)**. Scale bar=20 µm.

Avp immunolabelling in POA neurons (**Figure 5**) was absent in sections pre-adsorbed with Avp (**Figure 5B**), but not when pre-adsorbed with Oxt (**Figure 5C**). Subpopulations of POA neurons were identified based on their neuroanatomical positions in the rostral-caudal and ventral-dorsal axes as previously described (50). Avp-immunopositive neurons were found exclusively in the POA in periventricular locations. In the anterior POA, Cyp19a1b-expressing RGCs in the peripheral layer of the ventral telencephalon were observed in contact with the soma of parvocellular Avp-immunopositive cell bodies (**Figure 6**). In the posterior POA, probable contact points between Cyp19a1b-expressing RGC fibres lining the diencephalic ventricle and cell bodies of magnocellular and gigantocellular Avp-immunopositive neurons were observable indicating potential functional relationships (**Figure 7**).

## 4 Discussion

It was previously reported that female *cyp19a1b* ^-/-^ mutant zebrafish paired with WT males have a significantly longer latency to initiate spawning compared to WT females during dyadic sexual behavior assays (10). This is suggestive of an impairment in the ability of a female mutant to identify and/or assess a potential mate. Since *cyp19a1b* is exclusively expressed in RGCs in the teleost brain (38), these findings indicate that locally produced E2 in RGCs is likely diffusing to activate Esrs in nearby neurons to affect the expression of behaviorally relevant estrogen-regulated genes. Indeed, teleost RGCs have been previously shown to have the full complement of steroidogenic enzymes necessary for *de novo* steroidogenesis and the importance of RGC-derived steroids in the teleost brain for diverse functions, from regulating neurogenesis to various aspects of social behavior has been previously proposed (51). To identify the potential neuroendocrine mechanism underlying the behavioral impairment observed in the *cyp19a1b* ^-/-^ mutant females, we used ddPCR experiments to first quantify changes in the Esrs (*esr1*, *esr2a*, *esr2b*), the nonapeptides (*avp*, *oxt*), and the secretogranin 2s (s*cg2a*, *scg2b*). Telencephalon and hypothalamus samples were analyzed separately at two time points corresponding to during (10h00) and outside (14h00) the time of zebrafish reproductive activity. The findings from the ddPCR experiments revealed significant effects of *cyp19a1b* mutation on the expression levels of *esr*s, *avp*, and *oxt* in the hypothalamus.

The telencephalon and hypothalamus of *cyp19a1b* ^-/-^ mutant females had significantly lower Esr levels compared to WT females. Levels of *esr1* were 1.5 times higher in the hypothalamus of WT fish compared to *cyp19a1b* ^-/-^ mutants. This finding is supported by the previously observed lower brain E2 levels in mutant females (10) and the known positive estrogenic regulation of *esr1* due to the presence of an ERE in its promoter region. Time-dependent expression profiles of *esr1* were also observed. Levels were 1.6 times higher in the hypothalamus at 14h00 compared to 10h00, and 1.9 times higher levels specifically at 10h00 in the telencephalon of WT females compared to *cyp19a1b* ^-/-^ mutants. Lower levels of *esr2a* and *esr2b* were also identified in female *cyp19a1b* ^-/-^ mutants compared to WT females with region- and time-specific effects. Levels of *esr2a* were 1.7 times higher in the telencephalon of WT females compared to *cyp19a1b* ^-/-^ mutant females, whilst in the hypothalamus both *esr2a* and *esr2b* levels were 3.1 and 2.4 times higher, respectively, in WT females compared to mutants specifically at 14h00.

There has been surprisingly little study of the importance of brain Esr signalling for female teleost sexual behavior, outside of fertility assessments. Of the few studies conducted, no effects of *esr2a* mutation were observed on female medaka sexual behavior (52); however, *esr2b* mutation affected female sexual receptivity. In pairwise mating trials of medaka, female *esr2b* ^-/-^ mutants were unreceptive to males and therefore did not spawn eggs despite normal ovarian function (53). The study revealed that the female-biased sexual dimorphism in *esr2b*-expressing neurons in the ventral telencephalic area and the magnocellular/gigantocellular portion of the posterior POA decreased following water-borne aromatase inhibitor treatment, indicating that aromatase activity positively regulates brain *esr2b* levels. Medaka *esr2b* was shown to be positively estrogen-regulated using a luciferase reporter cell line in combination with E2 treatment. Together, these findings demonstrate that aromatase activity positively regulates Esr2b signalling levels in female medaka in brain regions important for sexual behavior. Our study findings suggest that brain aromatase activity likely regulates *esr2b* levels in the female zebrafish brain and that these effects may be linked to female sexual behavior. While in-depth study of *esr1* ^-/-^ mutant effects on female teleost sexual behavior have not been investigated, we observed that *esr1* levels were lower in the hypothalamus of *cyp19a1b* ^-/-^ mutants compared to WT females at the time of reproductive activity. Further study of Esr1 involvement in female zebrafish sexual behavior seem particularly well warranted given the similar behavioral phenotypes of female Aro KO and Esr1 KO mice that display increased rejection of male stud mice during sexual behavior assays (11, 13; 54). In zebrafish, all three nEsrs are expressed in neuroendocrine regions controlling female sexual behavior, paralleling *cyp19a1b* expression (55, 56). Our findings of lower *esr* levels in the telencephalon and hypothalamus of female *cyp19a1b* ^-/-^ mutants that have abnormal spawning behavior draws attention to the need for further study of *esr* ^-/-^ mutant effects on female sexual behavior.

Both *avp* and *oxt* were expressed at significantly lower levels in the hypothalamus of *cyp19a1b* ^-/-^ mutant females compared to WT fish. The hypothalamus of WT females had 1.8 times and 3.2 times higher levels of *avp* and *oxt*, respectively, compared to *cyp19a1b* ^-/-^ mutants. These findings are supported by other teleost studies demonstrating that E2 administration increases brain *avp* and/or *oxt* levels (28–31, 57, 58). The lower nonapeptides levels in the hypothalamus of female *cyp19a1b* ^-/-^ mutants is particularly interesting considering the important role of Avp and Oxt signalling in modifying the salience of social information processing in the brain to affect behavior (35). Recent studies of nonapeptide mutant lines have identified important roles of Avp and Oxt signalling for mate assessment. Female *avp* ^-/-^ mutant zebrafish displayed significantly reduced sexual receptivity compared to WT females during sexual interactions with WT males (36). In another study, *oxt* ^-/-^ and *oxtr1* ^-/-^ mutant Japanese medaka showed reduced social recognition of familiar males compared to WT females during sexual behavior assays (37). An impairment of sensory information processing that functions in mate identification and assessment, such as might occur by reduced *avp* and/or *oxt* levels, could represent a link to the behavioral phenotype observed in the *cyp19a1b* ^-/-^ mutant females during sexual behavior assays (10). Together, these findings suggest that the longer latency to initiate spawning behavior in the *cyp19a1b* ^-/-^ mutant females could be a consequence of impaired processing of sensory information due to reduced positive estrogenic regulation of Avp and/or Oxt signalling in the brain.

There were significant main effects of time on *scg2a* and *scg2b* levels in the telencephalon that confirm their high expression levels in the morning during the time at which zebrafish are sexually active. However, there were only minor differences in expression levels between the brains of *cyp19a1b* ^-/-^ mutant and WT females. For example, *scg2b* levels in the telencephalon of WT females were 1.2 times higher than those observed in *cyp19a1b* ^-/-^ mutants. Though these data indicate some change in *scg2b* levels, they are unlikely to explain the significant behavioral phenotype of the *cyp19a1b* ^-/-^ mutant females. Of note, *scg2a* ^-/-^ and *scg2b* ^-/-^ mutant female zebrafish are characterized by an ovulatory rather than behavioral dysfunction that underlies their impaired reproductive capacity (40). Therefore, the behavioral dysfunction in the *cyp19a1b* ^-/-^ mutant females seems unlikely to be directly associated with impaired SN signalling.

Based on the differences observed in the expression levels of genes between the morning and afternoon sampling times, we then assessed E2 brain content in *cyp19a1b* ^-/-^ mutants and WT females to determine if there were significant differences at the sampling time points. Female WT brains had 1.7 times higher E2 content compared to *cyp19a1b* ^-/-^ mutant brains confirming our previous study (10). Brain E2 content was 1.4 times higher in the afternoon compared to the morning. This difference in brain E2 content most likely represents E2 that crosses the blood-brain barrier from systemic circulation due to increased Cyp19a1a activity in the ovary at this time. Since no significant differences in ovarian E2 content (10) or serum E2 levels (43) were previously observed between *cyp19a1b* ^-/-^ mutants and WT females, the larger increase in brain E2 content from 10h00 to 14h00 in WT females compared to *cyp19a1b* ^-/-^ mutant females could represent the onset of brain *cyp19a1b* upregulation in WT females that does not occur in the *cyp19a1b* ^-/-^ mutants. Support for this hypothesis is provided by a previous gene expression study that identified a peak in ovarian *cyp19a1a* levels at the mid-point of the light phase that was followed by later increased brain *cyp19a1b* levels (59). This daily rhythm of gene expression is hypothesized to serve important functions in regulating reproductive activity in diurnal spawners like that observed in seasonally breeding species. In seasonally breeding teleosts, increased circulating E2 levels in females, driven by ovarian recrudescence and increased Cyp19a1a activity in the ovary, peak prior to the breeding season. This increases brain *cyp19a1b* levels and Cyp19a1b activity in the breeding months, due to the positive estrogenic regulation of *cyp19a1b* (60–63). The expected increase in brain *esr1* levels and E2 content in the afternoon was absent in female *cyp19a1b* ^-/-^ mutants. While speculative, it is possible that this altered expression profile is part of the mechanism underlying the behavioral impairment in female *cyp19a1b* ^-/-^ mutants.

We conducted acute intraperitoneal injections of teleost Avp and Oxt to see if either nonapeptide could rescue the behavioral phenotype in the *cyp19a1b* ^-/-^ mutant females. The nonapeptides were selected for study in the rescue experiment based on their known effects on social recognition and the findings that *avp* and *oxt* levels were ~45% and ~68% lower, respectively, in the hypothalamus of *cyp19a1b* ^-/-^ mutants compared to WT fish. Acute intraperitoneal Avp injections could rescue the time to the first oviposition event in female *cyp19a1b* ^-/-^ mutants. This behavioral rescue was receptor-dependent because antagonism of Avpr1a receptor signalling by co-administration of Manning Compound blocked the effect of Avp to advance oviposition in female mutants. In marked contrast, coadministration of the Oxtr antagonist L-368,899 had no effect on Avp action. This demonstrates that the rescue effects observed are specific to Avp signalling pathways.

These findings are supported by a previous study in female Asian stinging catfish (*Heteropneustes fossilis*), whereby low dosage 0.1 µg/g bw E2 intraperitoneal injections given daily for three days in ovariectomized females increased brain Avp levels (29). A functional relationship between E2 and Avp has also been previously observed in female round gobies (*Neogobius melanostomus*) during the spawning-capable phase whereby brain explants perfused *in vitro* with E2 released higher concentrations of Avp into the surrounding medium (31). Perfusion of brain explants with E2 increased Oxt release *in vitro*. Nonapeptide release induced by E2 could be blocked by administration of actinomycin D, demonstrating the dependence of E2 action on *de novo* RNA synthesis. It is interesting to note that Avp release also occurred rapidly, within 20 mins of E2 administration, suggesting an additional non-genomic pathway of estrogenic regulation. It would be interesting in a future study to measure the levels of bioactive nonapeptides in the brains of *cyp19a1b* ^-/-^ mutants to complement the findings in the current study. Indeed, previous studies in other teleosts have demonstrated links between oviposition and bioactive brain nonapeptide levels in the females (25, 64). It will also be important in future study to identify the potential additional contributions of Gper (the teleost mEsr) signalling to the observed behavioral phenotype in the *cyp19a1b* ^-/-^ mutants. Since significantly lower levels of nuclear *esr*s were identified in addition to *avp* in the hypothalamus of *cyp19a1b* ^-/-^ mutants compared to WT females, the current findings suggest at least a genomic regulatory mechanism underlying the behavioral phenotype observed in the mutant fish.

Since acute Avp injection uniquely rescued the delayed oviposition in *cyp19a1b* ^-/-^ mutants, we wanted to establish the neuroanatomical basis for Cyp19a1b-derived estrogenic communication with Avp neurons. To investigate the neuroanatomical proximity for brain-derived E2 induction of *avp* levels, we used immunohistochemistry with a teleost-validated Avp antibody and the Tg(*cyp19a1b*-GFP) zebrafish line (44). Close neuroanatomical proximities between Cyp19a1b-positive RGC fibres and Avp-immunopositive cell bodies were identified in the anterior and posterior parts of the POA. This could allow RGC-derived E2 to diffuse into nearby Avp-immunopositive neurons to regulate *avp* levels in the female zebrafish brain. A close association between Cyp19a1b-immunopositive RGC fibres and *avp*/Avp-expressing neurons in the POA has also been previously demonstrated in the orange-spotted grouper (*Epinephelus coioides*; 30) and the bluehead wrasse (*Thalassoma bifasciatum*; 65). In the orange-spotted grouper, *avp* colocalized with *esr1*, *esr2b* and *gper* in all three preoptic neuron populations- the parvocellular, magnocellular, and gigantocellular neurons, indicating the potential for direct estrogenic regulation of *avp* levels (30). In the bluehead wrasse, Cyp19a1b-positive RGC fibres were observed in close association to Avp-immunopositive cell bodies in the POA, indicating a potential functional relationship (65). Though the functional importance of the individual Avp subpopulations in teleost social behavior is currently unknown, evidence has suggested a potential role of gigantocellular neurons in female reproduction. A previous study in the half-spotted goby (*Asterropteryx semipunctata*) identified significantly high numbers of gigantocellular neurons in the peak-spawning period compared to the pre- and post-spawning periods, which was not seen similarly in the parvocellular or magnocellular neurons (22). These differences were also observed, however, in the non-spawning season and thus it is unclear whether the gigantocellular Avp-immunopositive neurons contribute to female reproduction. It will be important in future to identify if nEsrs colocalize with Avp in the female zebrafish brain to determine if there is a direct pathway for estrogenic regulation of *avp*. Future study should also identify which Avp-immunopositive subpopulations in the POA are important for the observed effects on female sexual behavior.

## 5 Conclusions

We have demonstrated that Avp rescues the behavioral phenotype of female *cyp19a1b* ^-/-^ mutant zebrafish. Our data indicate that lower brain E2 levels in the *cyp19a1b* ^-/-^ mutant females reduces Avp signalling in the hypothalamus leading to an increased latency to the first oviposition event during pairwise mating trials. Contact points between Cyp19a1b-expressing RGCs and Avp-immunopositive cell bodies in the POA identify the neuroanatomical proximity and region of interest for locally produced E2 to regulate *avp* levels. Rescue of delayed oviposition with a single injection of Avp indicates the importance of the RGC-Avp network in the regulation of female behavior. Further study will be needed to identify nEsr and Avp colocalization and the neuronal subpopulations in the POA responsible for the observed effects on female spawning behavior.

## Data availability statement

The raw data supporting the conclusions of this article will be made available by the authors, without undue reservation.

## Ethics statement

The animal study was approved by University of Ottawa Protocol Review Committee. The study was conducted in accordance with the local legislation and institutional requirements.

## Author contributions

KS: Conceptualization, Data curation, Formal analysis, Investigation, Methodology, Visualization, Writing – original draft, Writing – review & editing. CL: Methodology, Writing – review & editing. XL: Methodology, Writing – review & editing. VT: Conceptualization, Funding acquisition, Resources, Supervision, Writing – review & editing.
